# Mendelian randomization analysis of 34,497 German Holstein cows to infer causal associations between milk production and health traits

**DOI:** 10.1186/s12711-024-00896-5

**Published:** 2024-04-08

**Authors:** Helen Schneider, Valentin Haas, Ana-Marija Krizanac, Clemens Falker-Gieske, Johannes Heise, Jens Tetens, Georg Thaller, Jörn Bennewitz

**Affiliations:** 1 Institute of Animal Science, University of Hohenheim, 70599 Stuttgart, Germany; 2 Department of Animal Sciences, University of Göttingen, 37077 Göttingen, Germany; 3 Vereinigte Informationssysteme Tierhaltung w.V. (VIT), 27283 Verden, Germany; 4 Institute of Animal Breeding and Husbandry, Christian-Albrechts University of Kiel, 24098 Kiel, Germany

## Abstract

**Background:**

Claw diseases and mastitis represent the most important health issues in dairy cattle with a frequently mentioned connection to milk production. Although many studies have aimed at investigating this connection in more detail by estimating genetic correlations, they do not provide information about causality. An alternative is to carry out Mendelian randomization (MR) studies using genetic variants to investigate the effect of an exposure on an outcome trait mediated by genetic variants. No study has yet investigated the causal association of milk yield (MY) with health traits in dairy cattle. Hence, we performed a MR analysis of MY and seven health traits using imputed whole-genome sequence data from 34,497 German Holstein cows. We applied a method that uses summary statistics and removes horizontal pleiotropic variants (having an effect on both traits), which improves the power and unbiasedness of MR studies. In addition, genetic correlations between MY and each health trait were estimated to compare them with the estimates of causal effects that we expected.

**Results:**

All genetic correlations between MY and each health trait were negative, ranging from − 0.303 (mastitis) to − 0.019 (digital dermatitis), which indicates a reduced health status as MY increases. The only non-significant correlation was between MY and digital dermatitis. In addition, each causal association was negative, ranging from − 0.131 (mastitis) to − 0.034 (laminitis), but the number of significant associations was reduced to five nominal and two experiment-wide significant results. The latter were between MY and mastitis and between MY and digital phlegmon. Horizontal pleiotropic variants were identified for mastitis, digital dermatitis and digital phlegmon. They were located within or nearby variants that were previously reported to have a horizontal pleiotropic effect, e.g., on milk production and somatic cell count.

**Conclusions:**

Our results confirm the known negative genetic connection between health traits and MY in dairy cattle. In addition, they provide new information about causality, which for example points to the negative energy balance mediating the connection between these traits. This knowledge helps to better understand whether the negative genetic correlation is based on pleiotropy, linkage between causal variants for both trait complexes, or indeed on a causal association.

## Background

In farm animals, the negative side effects of a high production performance on animal health has raised concerns regarding the ecological footprint of livestock, animal welfare and the farmers’ economy [1–3]. For dairy cattle, this implies that an increased milk yield is accompanied by increased incidences of diseases, such as mastitis or claw diseases and fertility problems. With the aim to identify whether this issue is genetically determined or related to management deficiencies, geneticists have repeatedly estimated the genetic correlations between milk production and health traits. Genetic correlations can arise because of several reasons, such as linkage between causal variants for different traits or horizontal pleiotropy [4, 5], i.e., that a genetic variant affects more than one trait. Thus, horizontal pleiotropy has to be separated from vertical pleiotropy, which describes the phenomenon that a genetic variant affects traits on the same causal pathway [6]. In addition, genetic correlations can indicate causality [4]. However, a detailed inference about causal associations from genetic correlations is not possible [7, 8].

The gold standard to assess the causal effect of an exposure (e.g., a treatment) on an outcome variable (e.g., a disease trait) is the randomized control trial (RCT). Here, individuals are randomly assigned (“randomized”) to groups categorized by exposure (e.g., treatment/control), which allows to differentiate between the effect of the exposure itself and the effect of potential confounding factors that can also evoke a causal association between the exposure and the outcome variable [4]. In humans, RCT allowed to confirm that low-density lipoprotein (LDL) cholesterol has an effect on coronary heart disease [9, 10], which was previously suggested in an observational study [11]. In dairy cattle, RCT were performed, e.g., to investigate the economic benefit of different voluntary waiting periods [12], the effect of homeopathy and teat sealers on mastitis [13] or the effects of different preventive methods for ketosis, such as the reduction in milking frequency [14]. Nevertheless, RCT are not feasible to assess causal associations in every setting. For some queries, they might be expensive, impracticable, or even unethical. In addition, it is impossible to test the association of multiple interventions on an outcome variable in a RCT [4, 15]. An alternative is the so-called Mendelian randomization (MR) analysis, where genetic variants [instrumental variables (IV)] are exploited for randomization. The advantage of using genetic variants is that they are present from conception and remain (approximately) unchanged during life. Hence, they are expected to be free from confounding factors [4, 15, 16]. Earlier studies performed MR analyses by using one or only a few genetic variants as IV [17, 18], which might result in an underpowered MR if the variants have only small effect sizes. However, this can be outperformed by analysing multiple variants jointly in one analysis [15, 19], e.g., by performing a summary-statistics-based MR (SMR, [20]), where summary statistics from genome-wide association studies (GWAS) are used. Recently, this has also been extended to a generalized SMR (GSMR) [15], where, linkage disequilibrium (LD) among the variants is taken into account since LD results in biased MR estimates [19]. Selection of the genetic variants that will be adequate and valid IV for obtaining unbiased MR estimates relies on three assumptions [8]: (1) the relevance assumption, which implies that a variant is strongly associated with the exposure variable, (2) the independence assumption that defines a valid IV as sharing no common cause with the outcome variable, and (3) the exclusion restriction assumption implying that the IV affect the outcome only via the exposure, i.e., not via horizontal pleiotropy [8, 16].

A broad range of MR analyses have been performed in human genetics, where they are facilitated by the public availability of large sample size GWAS summary statistics. These MR were able to identify causal relationships e.g., between body mass index and coronary heart disease [21], vitamin D and mortality [22] or alcohol consumption and heart disease [23]. In contrast, to date only a few MR analyses have been performed in livestock genetics. These have investigated e.g., the causal association between average daily milk yield and resilience indicator traits [24] or between several stature and milk production traits and some functional traits, such as somatic cell count and fertility [25]. So far, no study has examined the causal association of milk production with health traits in dairy cows. Thus, our aim was to fill this gap by investigating the causal association of milk yield (MY) with six claw health traits and mastitis (MAS). Towards this aim, we used a large sample of German Holstein cows with imputed whole-genome sequence (WGS) data, consisting of 17 million sequence variants. The study was split into three parts. First, we estimated heritabilities and genetic correlations based on a 50K single nucleotide polymorphism (SNP) chip data, to compare the observational and causal associations that we expect to identify in our sample. Then, we performed a GWAS for each trait to generate summary statistics. Finally, these summary statistics were used in the last step, which consisted of a set of MR analyses with MY as the exposure variable and each of the health traits as the outcome variable. Here, our aim was to investigate the connection between these traits in more detail. It is thought that this knowledge will reveal whether the negative side effects of high milk production in dairy cattle are due to horizontal pleiotropy, confounding factors or indeed to a causal effect of milk yield on health traits.

## Methods

### Material

In this study, we analyzed 34,497 German Holstein cows, which had their first lactation between 2015 and 2020. This dataset is a subset of a larger dataset, provided by the national computing center in Germany (VIT, Vereinigte Informationssysteme Tierhaltung w.V., Verden, Germany), that we filtered as described in Schneider et al. [26]. De-regressed proofs (DRP) with homogeneous accuracies of about 0.75 were available for seven health traits. They are based on on-farm recording by farmers, veterinarians and claw trimmers of disease cases. In addition to mastitis (MAS), the recorded health traits comprised the following claw diseases: claw ulcers (CU), digital dermatitis (DD), interdigital hyperplasia (IH), laminitis (LAM), white line disease (WL) and digital phlegmon (PH). It is important to mention that a lower DRP for a health trait indicates an unfavorable health status. In addition, DRP were available for milk yield (MY). An overview of the number of individuals with DRP for each trait is in Table 1.

**Table 1:** Overview of the traits and the number of individuals with deregressed proofs for each trait

Trait	Trait abbreviation	Number of individuals	${{{h}}}^{2}$ (SE)
Claw ulcers	CU	27,012	0.153 (0.007)
Digital dermatitis	DD	30,056	0.175 (0.007)
Digital phlegmon	PH	26,437	0.098 (0.006)
Interdigital hyperplasia	IH	30,968	0.153 (0.007)
Laminitis	LAM	30,997	0.176 (0.007)
Mastitis	MAS	33,298	0.133 (0.006)
Milk yield	MY	34,497	0.436 (0.008)
White line disease	WL	30,973	0.139 (0.007)

${h}^{2}$: heritability estimates of the traits with their standard errors (SE).

### Genotypes

For all 34,497 cows, 50K chip and imputed WGS data were available. The 50K chip data were provided by the VIT and the imputation is described in Križanac et al. [27]. After filtering out the SNPs located on the sex chromosomes and those with a minor allele frequency (MAF) lower than 0.01, 44,126 SNPs of the 50K chip remained for the analysis. For the imputed WGS variants, we applied the same filtering criteria but set the MAF threshold to 0.05. Moreover, the dosage R-squared parameter (DR2) that measures the squared correlation between the true and the estimated allele dosage [28] was applied to assess the quality of the imputed WGS data. Variants were filtered out if the DR2 was lower than 0.75 [27] and, in total, 16,882,734 imputed WGS variants remained for further analysis.

### Statistical analysis

First, we performed a univariate variance component estimation for each trait with the following mixed linear model using the GCTA software version 1.92.3 beta3 [29]:

1 $$\mathbf{y}={\mathbf{1}}{\mu} +\mathbf{Z}\mathbf{g}+\mathbf{e},$$

where, $\mathbf{y}$ is the vector of the DRP of each animal, **1** is a vector of ones, *µ* denoting the mean, $\mathbf{e}$ is the vector of the residuals and $\mathbf{g}$ is the vector of the polygenic term with $\mathbf{Z}$ as the corresponding incidence matrix that links the polygenic term to the trait records. It was assumed that both $\mathbf{g}$ and $\mathbf{e}$ follow a normal distribution with $\mathbf{g} \sim N(0, {\mathbf{G}}_{\mathbf{50K}}{\sigma }_{g}^{2})$ and $\mathbf{e} \sim N(0, \mathbf{I}{\sigma }_{e}^{2})$, where $\mathbf{I}$ is the identity matrix and ${\mathbf{G}}_{\mathbf{50K}}$ the additive genetic relationship matrix (GRM) of the 50K chip. The construction of the GRM involved all 34,497 animals and its computation was performed using GCTA [30].

Then, we applied the following bivariate model to estimate the variance components for seven trait-combinations, which all consisted of MY (${\mathbf{y}}_{1})$ and one health trait (${\mathbf{y}}_{2}$):

2 $$\left[\begin{array}{c}{\mathbf{y}}_{\mathbf{1}}\\ {\mathbf{y}}_{\mathbf{2}}\end{array}\right]=\left[\begin{array}{c}{\mathbf{1}\mu }_{1}\\ {\mathbf{1}}{\mu }_{2}\end{array}\right]+\left[\begin{array}{cc}{\mathbf{Z}}_{\mathbf{1}}& 0\\ 0& {\mathbf{Z}}_{\mathbf{2}}\end{array}\right]\left[\begin{array}{c}{\mathbf{g}}_{\mathbf{1}}\\ {\mathbf{g}}_{\mathbf{2}}\end{array}\right]+\left[\begin{array}{c}{\mathbf{e}}_{\mathbf{1}}\\ {\mathbf{e}}_{\mathbf{2}}\end{array}\right],$$

where ${\mu }_{1}$ and ${\mu }_{2}$ denote the means of ${\mathbf{y}}_{\mathbf{1}}$ and ${\mathbf{y}}_{\mathbf{2}}$ with ${\mathbf{Z}}_{\mathbf{1}}$ and ${\mathbf{Z}}_{\mathbf{2}}$ being the corresponding incidence matrices. The variance–covariance-matrix of the respective polygenic (${\mathbf{g}}_{\mathbf{1}}$ and ${\mathbf{g}}_{\mathbf{2}}$) and residual (${\mathbf{e}}_{\mathbf{1}}$ and ${\mathbf{e}}_{\mathbf{2}}$) terms was modeled as:

3 $$var\left[\begin{array}{c}{\mathbf{g}}_{\mathbf{1}}\\ {\mathbf{g}}_{\mathbf{2}}\\ \begin{array}{c}{\mathbf{e}}_{\mathbf{1}}\\ {\mathbf{e}}_{\mathbf{2}}\end{array}\end{array}\right]=\left[\begin{array}{cc}{\mathbf{G}}_{\mathbf{50K}}{\sigma }_{g1}^{2}& {\mathbf{G}}_{\mathbf{50K}}{\sigma }_{g12}\\ {\mathbf{G}}_{\mathbf{50K}}{\sigma }_{g12}& {\mathbf{G}}_{\mathbf{50K}}{\sigma }_{g2}^{2}\\ \begin{array}{c}0\\ 0\end{array}& \begin{array}{c}0\\ 0\end{array}\end{array} \begin{array}{cc}0& 0\\ 0& 0\\ \begin{array}{c}\mathbf{I}{\sigma }_{e1}^{2}\\ {\mathbf{I}\sigma }_{e12}\end{array}& \begin{array}{c}{\mathbf{I}\sigma }_{e12}\\ {\mathbf{I}\sigma }_{e2}^{2}\end{array}\end{array}\right].$$

We calculated the heritabilities (${h}^{2}$) and genetic correlations (${r}_{g}$) by applying standard notations.

### Genome-wide association study

The imputed WGS data were used to perform a GWAS for each trait using mixed linear models that are implemented in GCTA [29]. Three models were applied that used different methods to account for population structure. This was done to ensure sufficient power to detect trait-associated signals but also to avoid inflated type I errors. The first method included a polygenic term that implemented the full GRM across all chromosomes (known as MLMA in GCTA applications). However, the MLMA method suffers from a reduced power due to the double-fitting of the candidate variants, both in the GRM and as fixed effects. Thus, we also applied the so-called “leave-one-chromosome-out” (LOCO) method. Here, the GRM is adjusted such that the chromosome that harbors the candidate variant was excluded from the calculation of the GRM [31]. However, it has been shown that the LOCO method can generate substantial genomic inflation due to the underestimated relationship between individuals because of the missing chromosome in the LOCO-adjusted GRM (e.g., [26, 32, 33]). Thus, in addition, we applied the PC_CHR method that is an extension of the LOCO approach with the aim to account for the reduced informativity of the LOCO-adjusted GRM. GCTA was applied to compute 20 principal components (PC) for each chromosome using the 50K chip data. Then, the PC were included as covariates in the model. We applied the PC on a chromosomal level since, in contrast to the PC on a genome-wide level, they do not include overlapping information with the GRM [33]. All three methods applied the following mixed linear model that is implemented in GCTA:

4 $$\mathbf{y}=\mathbf{X}\mathbf{b}+\mathbf{g}+\mathbf{e},$$

where $\mathbf{y}$ is the vector of the DRP that were standardized to $\mathbf{y} \sim N(\mathrm{0,1})$ to facilitate the interpretation of variant effects. $\mathbf{b}$ is the vector of the mean and the fixed effects that include the effects of the variants and the 20 chromosomal PC for the PC_CHR method. $\mathbf{X}$ denotes the incidence matrix that links $\mathbf{b}$ to the number of allele copies of the variants and of trait records to the covariate effects. $\mathbf{g}$ and $\mathbf{e}$ are the vectors of the polygenic and residual terms, respectively, and followed a normal distribution with $\mathbf{g}\boldsymbol{ }\sim N(0,\boldsymbol{ }{\mathbf{G}}_{\mathbf{50K}}{\sigma }_{g}^{2})$ and $\mathbf{e} \sim N(0, \mathbf{I}{\sigma }_{e}^{2})$, where either the complete or the LOCO-adjusted GRM was applied. Then, we calculated the genomic inflation factor ${\uplambda }_{{\text{GC}}}$ to assess the inflation of type I errors by using the p-values of the variants to obtain the ratio of the median of the observed chi-square test statistics with one degree of freedom to the corresponding expected median [32].

### Generalized summary-data based Mendelian randomization

In the subsequent MR analysis, we investigated the causal relationship between MY and each of the seven health traits. For the health traits, we used the summary statistics generated with the MLMA approach and for MY, we used the summary statistics generated with the PC_CHR method, because these summary statistics resulted in the inflation factor values that were closest to 1. Our decision relates to the narrow ridge that exists between a lack of power to detect causal associations in our dataset due to a deflated summary statistic, and an increased type 1 error rate in the MR analysis due to a substantial inflation in the summary statistics [26].

The MR analysis was carried out using the GSMR method [15] that is also implemented in GCTA [29]. A detailed description of the method is in Zhu et al. [15], but we will give a brief explanation below. Following the suggestions of Zhu et al. [15], the top-associated (${p}_{GWAS, MY}< {5*10}^{-8}$) and quasi-independent variants after clumping (${r}^{2}<0.05$) within a window of 1 Mb were selected as IV. We set the number of IV required to perform a MR analysis to a minimum of ten.

We defined MY as the exposure ($x$) and each of the seven health traits as the outcome ($y$) variable. Then, ${\widehat{b}}_{zx(i)}$ denotes the effect of the $i$-th IV ($z$) on MY and ${\widehat{b}}_{zy(i)}$ the effect of $z$ on $y$. The key-point and central output of the GSMR is the MR estimate ${\widehat{b}}_{xy}$ that indicates the causal effect of the exposure on the outcome variable. For the $i$-th variant, it is calculated as:

5 $${\widehat{b}}_{xy(i)}= \frac{{\widehat{b}}_{zy(i)} }{{\widehat{b}}_{zx(i)}}.$$

For all IV with $m$ as the number of IV, ${\widehat{\mathbf{b}}}_{\mathbf{x}\mathbf{y}}$ is then defined as:

6 $${\widehat{\mathbf{b}}}_{\mathbf{x}\mathbf{y}}=\left\{{\widehat{b}}_{xy\left(1\right)}, {\widehat{b}}_{xy\left(2\right)}, ..., {\widehat{b}}_{xy\left(m\right)}\right\},$$

and follows a normal distribution with ${\widehat{\mathbf{b}}}_{\mathbf{x}\mathbf{y}}\sim N\left({\mathbf{b}}_{\mathbf{x}\mathbf{y}},\mathbf{V}\right)$. Thereby, $\mathbf{V}$ denotes the variance–covariance matrix of ${\widehat{\mathbf{b}}}_{\mathbf{x}\mathbf{y}}$ that contains the LD correlation between the IV [15]. Including horizontal pleiotropic variants in the analysis would violate one of the initially mentioned IV assumptions, i.e., the exclusion restriction assumption [6]. Thus, the GSMR approach includes the heterogeneity-in-dependent-instruments (HEIDI) method that detects and removes horizontal pleiotropic variants [15]. The HEIDI method is based on the idea that, in the absence of horizontal pleiotropy and if the effect of $x$ on $y$ is causal, ${\widehat{b}}_{xy}$ is only based on the effect of $z$ on $y$ via $x$ and will be identical for any IV. Briefly, the method consists in computing for each IV ${z}_{i}$, the deviation ${d}_{i}$ of ${\widehat{b}}_{xy(i)}$ from ${\widehat{b}}_{xy(i)}$ at a target variant (${\widehat{b}}_{xy(top)}$). A target IV is defined as the IV that shows the strongest association with the exposure variable (MY) in the third quintile of the ordered distribution $- lo{g_{10}}(p{\text{-}}value)$ of ${\widehat{b}}_{zx}$. This procedure was applied to define the target IV, although the power to detect a horizontal pleiotropic outlier increases, as the association with the exposure is stronger. However, variants with a strong association are also likely to be horizontal pleiotropic variants [15]. Thus, it would not be straightforward to use the IV that shows the strongest association with the exposure variable as target variant in order to detect horizontal pleiotropic outliers. Then, ${d}_{i}$ at the $i$-th IV is defined as ${d}_{i}= {b}_{xy(i)}- {b}_{xy(top)}$ with:

7 $$var\left({\widehat{d}}_{\left(i\right)}\right)=var\left({\widehat{b}}_{xy\left(i\right)}-{\widehat{b}}_{xy\left(top\right)}\right),$$

where LD among the IV is again considered, which implies that the HEIDI method filters for the LD that was not already removed by the clumping step [15]. In order to obtain the statistical significance of the MR estimate (${\widehat{b}}_{xy}$) and the deviation of ${\widehat{b}}_{xy(i)}$ from ${\widehat{b}}_{xy(top)}$, the test statistics ${T}_{GSMR}$ and ${T}_{HEIDI}$ were applied with:

8 $${T}_{GSMR}= \frac{{\widehat{b}}_{xy}^{2}}{var({\widehat{b}}_{xy})},$$

and

9 $${T}_{HEIDI}= \frac{{\widehat{d}}_{i}^{2}}{var({\widehat{d}}_{i})}.$$

They followed a Chi-square distribution with one degree of freedom. We chose two thresholds to declare a causal effect ${\widehat{b}}_{xy}$ as significant, i.e., a Bonferroni corrected threshold of 0.05, corrected for seven tests, (this corresponds to an experiment-wide significance) and one, without correction, of 0.05 (nominal significance). Horizontal pleiotropic outliers were defined as IV that significantly (${p}_{HEIDI}= 0.01$) deviate from homogeneity [15]. In this study, we applied two MR analyses, one that included the HEIDI method (HEIDI) and one that did not (noHEIDI). This was done to assess the contribution of horizontal pleiotropic variants to the putative causal association between the traits.

## Results

### Estimation of variance components

The heritability estimates of the DRP were moderate to low for the health traits and high (${h}^{2}=$ 0.439) for MY. Concerning the health traits, LAM had the highest heritability (${h}^{2}=$ 0.176) and PH the lowest (${h}^{2}=$ 0.098). All health traits were negatively correlated with MY. We found a negative high correlation between MY and MAS (${r}_{g}$ = − 0.303). The weakest correlation was between MY and DD (${r}_{g}$ = − 0.019) (Table 2). A genetic correlation was declared to be significantly different from zero if the absolute value of the estimate was at least twice the corresponding standard error. Following this definition, only the trait-combination consisting of DD and MY did not reach significance (${r}_{g}$ = − 0.019, SE = 0.026).

### Genome-wide association study

For the health traits, the genomic inflation factors were equal to 1.010 (MAS), 1.012 (IH), 1.015 (CU), 1.016 (DD), 1.031 (WL), 1.032 (PH) and 1.039 (LAM) using the MLMA method. These values were close to 1, which is why no other GWAS method was applied. In contrast, using the MLMA method, we found a strong deflation (${\uplambda }_{GC}$ = 0.900) for MY that became a strong inflation (${\uplambda }_{GC}$ = 15.157) when the LOCO method was applied. Thus, the summary statistics generated with the PC_CHR approach (${\uplambda }_{GC}$ = 1.569) was subsequently used in the GSMR analysis.

### Generalized summary-data based Mendelian randomization

After filtering for variants in high LD, 105 variants remained as IV for the GSMR analysis of MY and the seven health traits. In addition, three variants were removed by the HEIDI filtering step in the analysis of MY and MAS, two in the analysis of MY and DD and one in the analysis of MY and PH. Four of these variants were located on *Bos taurus* (BTA) autosome 6, one on BTA14 and one on BTA19 (Table 3). The effects of almost all the horizontal pleiotropic variants were in the same direction for MY and the health trait, with only the two variants on BTA6 that affect MY and MAS showing an antagonistic effect.

**Table 3:** Horizontal pleiotropic variants that were identified by the HEIDI method

Chr	Position	Milk yield		Health trait		
		$b$ (SE)	${p}_{GWAS}$	Trait	$b$ (SE)	${p}_{GWAS}$
6	82,842,199	0.055 (0.007)	< 0.0001	MAS	− 0.044 (0.012)	0.0002
6	85,526,144	− 0.110 (0.015)	< 0.0001	DD	− 0.055 (0.021)	0.0087
6	87,304,351	0.112 (0.008)	< 0.0001	MAS	− 0.060 (0.011)	< 0.0001
6	87,719,366	0.093 (0.013)	< 0.0001	DD	0.047 (0.020)	0.0177
14	6,054,352	0.110 (0.011)	< 0.0001	PH	0.038 (0.016)	0.0032
19	17,893,592	0.078 (0.013)	< 0.0001	MAS	0.052 (0.018)	0.0200

Shown are the chromosome (Chr) and position (in bp) of the variant, together with the effect size ($b$), standard error (SE) and p-value (${p}_{GWAS}$) from the association study for milk yield and the respective health trait

**Fig. 1:**
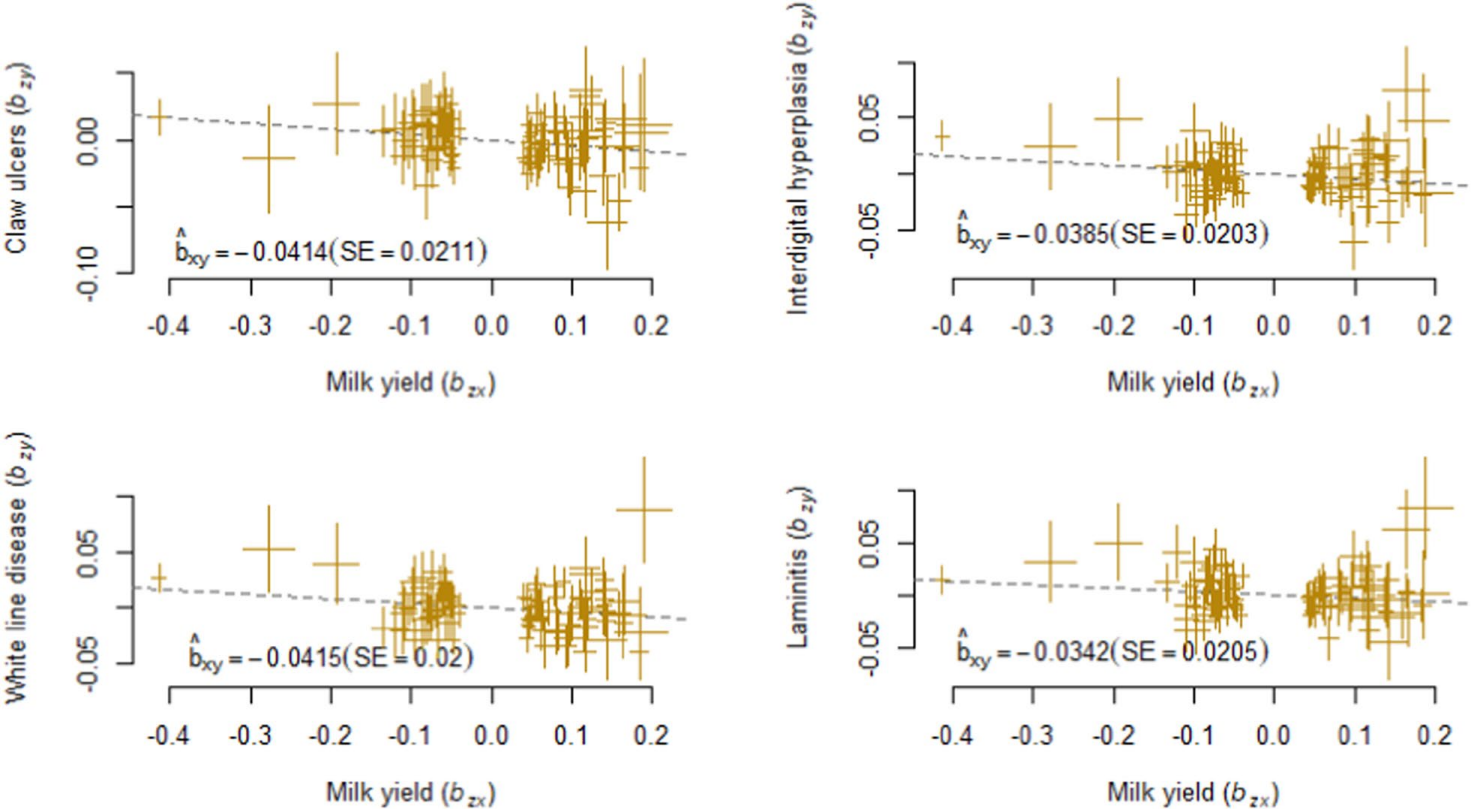
GSMR analysis of the respective health trait and milk yield. Shown are the plots of the effect sizes. For these trait combinations, no difference was observed between the analyses that did and the one that did not exclude horizontal pleiotropic variants

**Fig. 2:**
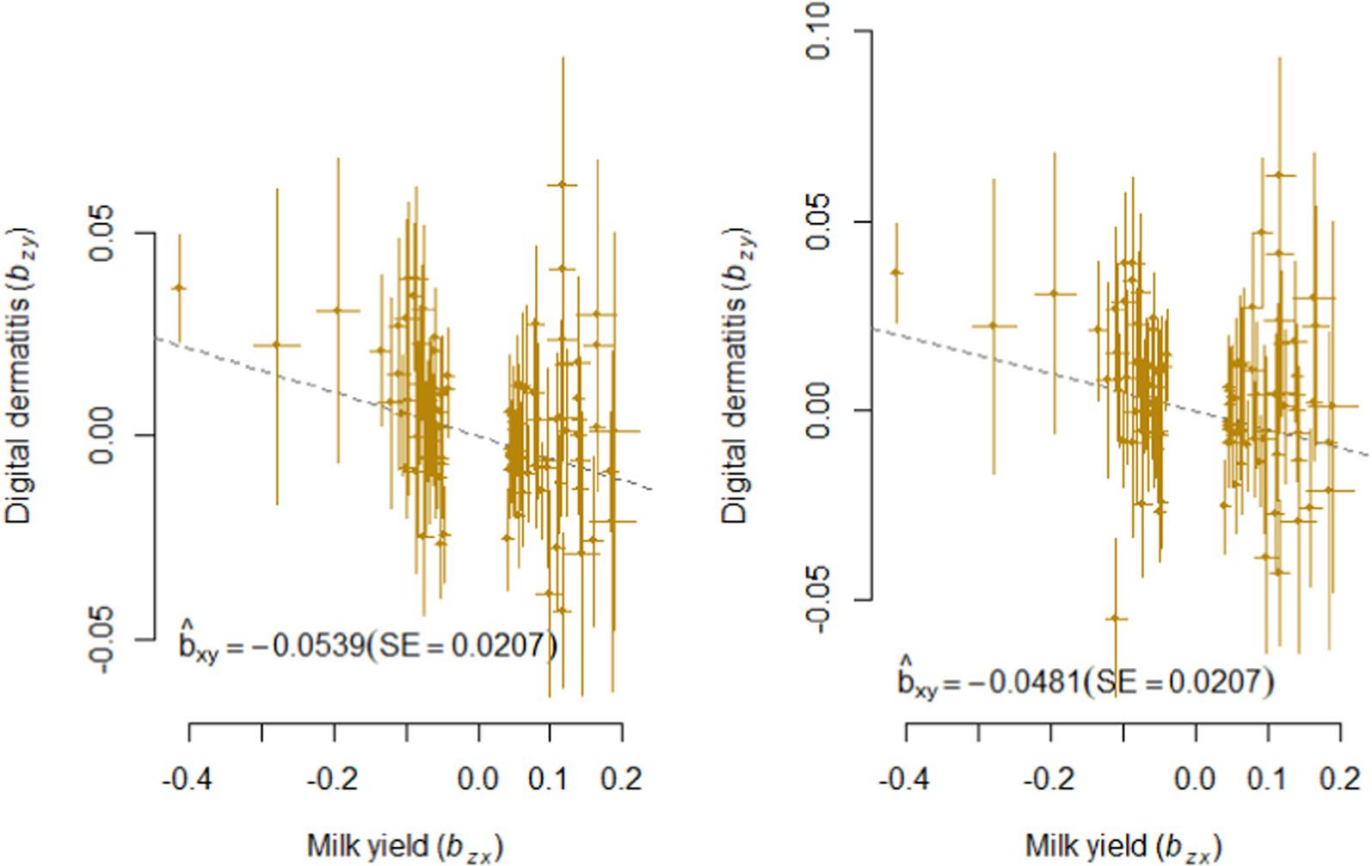
GSMR analysis of digital dermatitis and milk yield. Shown are the plots of the effect sizes for the analysis with (left) and without (right) filtering for horizontal pleiotropic variants

**Fig. 3:**
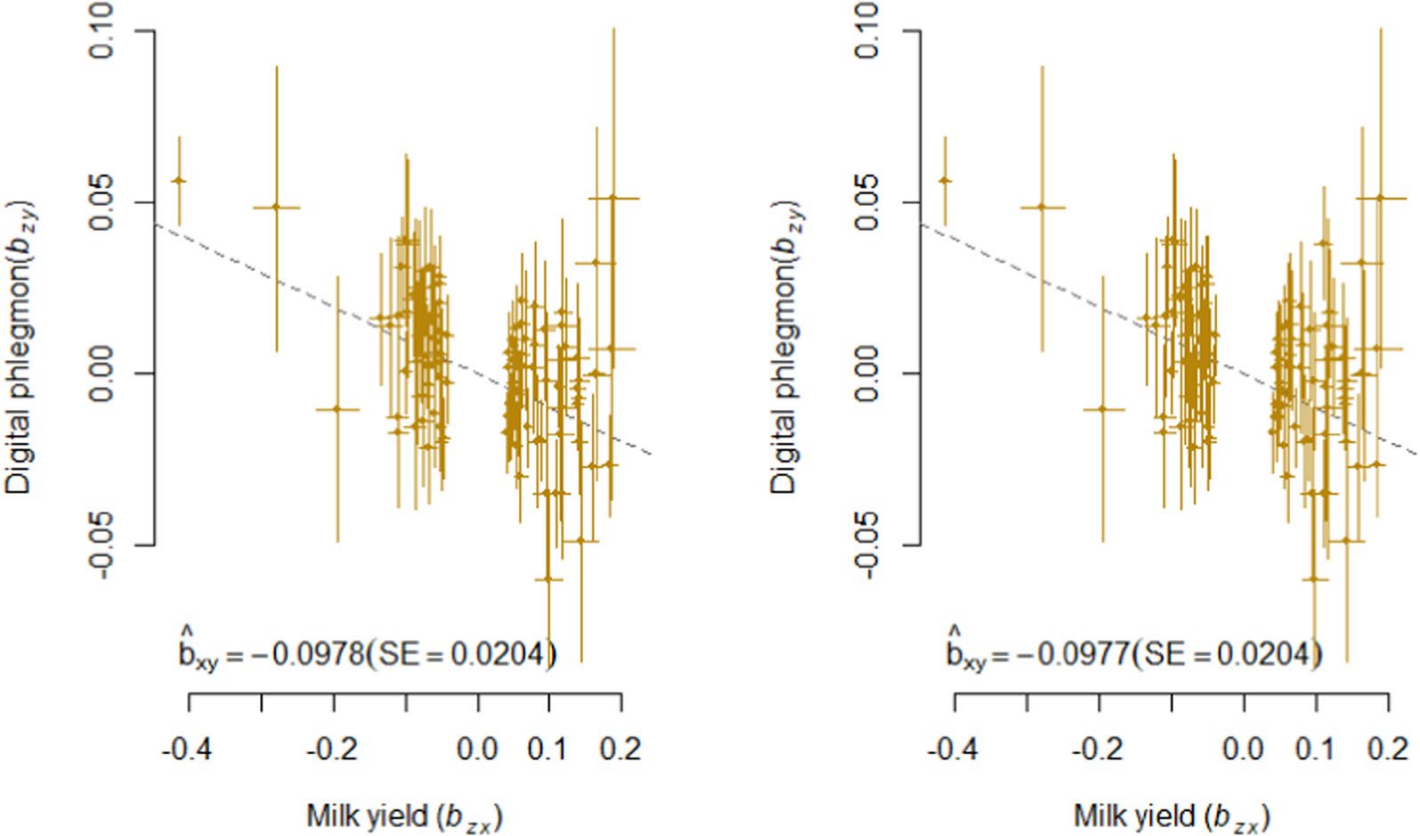
GSMR analysis of digital phlegmon and milk yield. Shown are the plots of the effect sizes for the analysis with (left) and without (right) filtering for horizontal pleiotropic variants

**Fig. 4:**
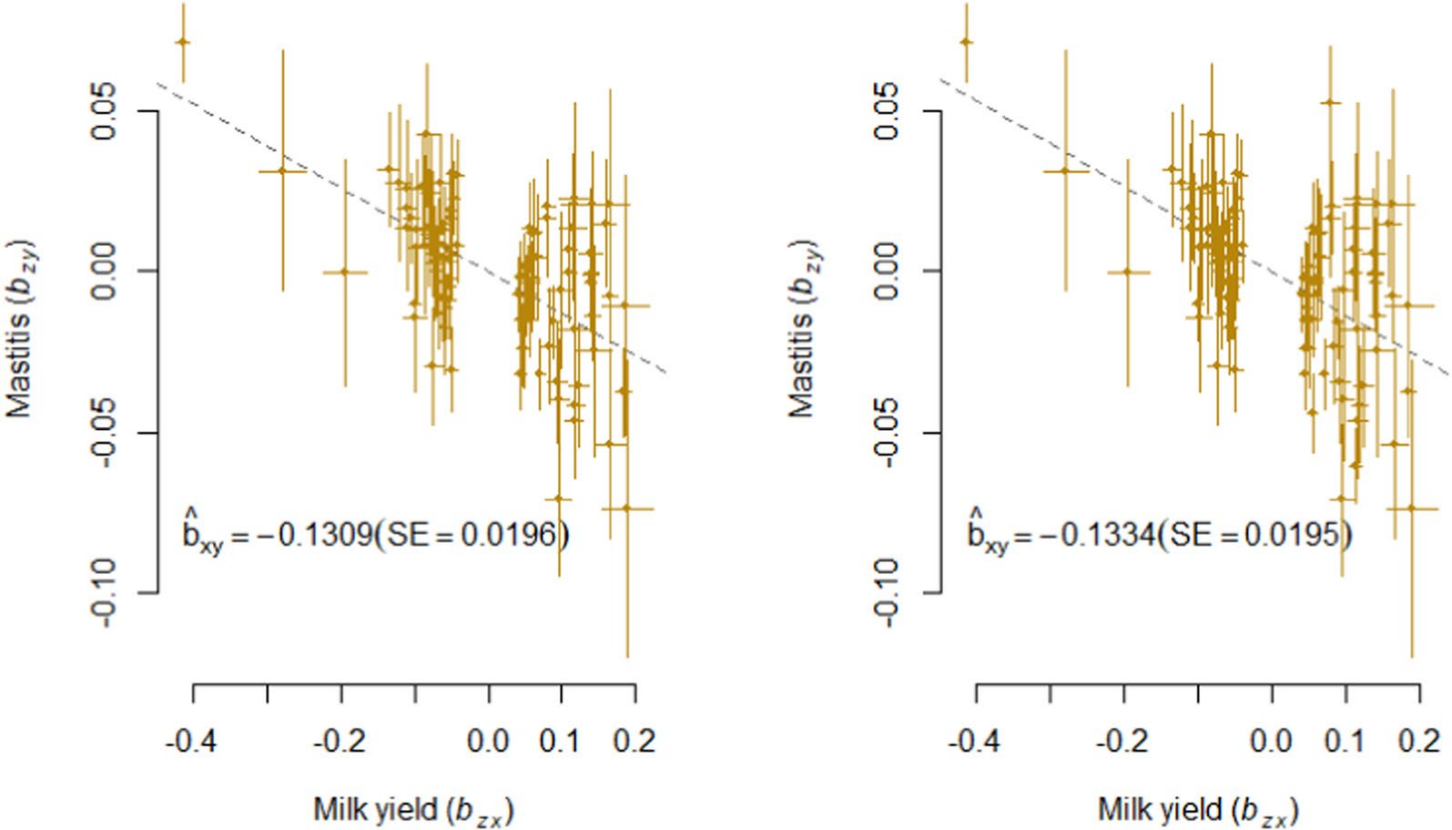
GSMR analysis of mastitis and milk yield. Shown are the plots of the effect sizes for the analysis with (left) and without (right) filtering for horizontal pleiotropic variants

Regarding the causal associations, the causal effects ${\widehat{b}}_{xy}$ of MY on any health trait were negative, which indicates a decrease in the DRP for the traits when the DRP for MY increases. In the HEIDI analysis, ${\widehat{b}}_{xy}$ ranged from − 0.133 (MAS) to − 0.034 (LAM) and in the noHEIDI analysis from − 0.131 (MY) to − 0.034 (LAM) (Table 2 and Figs. 1, 2, 3 and 4).

**Table 2:** Genetic correlations and causal associations between milk yield and the respective health trait

Health trait	${r}_{g}$ (SE)	HEIDI			noHEIDI		
		${\widehat{b}}_{xy}$ (SE)	${p}_{GSMR}$	N	${\widehat{b}}_{xy}$ (SE)	${p}_{GSMR}$	N
Claw ulcers	− 0.069 (0.028)	− 0.041 (0.021)	0.0494	105	− 0.041 (0.021)	0.0494	105
Digital dermatitis	− 0.019 (0.026)	− 0.054 (0.021)	0.0094	103	− 0.048 (0.021)	0.0200	105
Digital phlegmone	− 0.182 (0.032)	− 0.098 (0.020)	< 0.0001	104	− 0.098 (0.020)	< 0.0001	105
Interdigital hyperplasia	− 0.106 (0.027)	− 0.039 (0.020)	0.0577	105	− 0.039 (0.020)	0.0577	105
Laminitis	− 0.082 (0.040)	− 0.034 (0.021)	0.0577	105	− 0.034 (0.021)	0.0577	105
Mastitis	− 0.303 (0.026)	− 0.131 (0.020)	< 0.0001	102	− 0.133 (0.020)	< 0.0001	105
White line disease	− 0.088 (0.035)	− 0.042 (0.020)	0.0381	105	− 0.042 (0.020)	0.0381	105

Shown are the genetic correlations ($${r}_{g})$$ and effect sizes ($${\widehat{b}}_{xy}$$) from the GSMR analysis together with their standard errors (SE) as well as the p-values ($${p}_{GSMR}$$) and the number of instrumental variables (N) for the GSMR analysis. The results of the GSMR analysis are shown for the analysis with (HEIDI) and without (noHEIDI) applying the HEIDI method

In both analyses, we found a nominal significant causal effect of MY on each health trait except for LAM and IH and an experiment-wide significant causal effect of MY on PH and MAS. The causal effect of MY on DD almost reached experiment-wide significance (${p}_{GSMR}$ = 0.0094) in the HEIDI analysis. Here, ${\widehat{b}}_{xy}$ was − 0.054 and slightly lower (${\widehat{b}}_{xy}$ = − 0.048) when the horizontal pleiotropic variants were not removed (Table 2; Fig. 2). For MAS and PH, there was no obvious difference between the HEIDI and the noHEIDI analysis (Table 2 and Figs. 3 and 4).

## Discussion

Currently, knowledge about the relationship between milk production and health traits in dairy cattle is mostly acquired by estimating genetic correlations [2, 3]. However, there is a substantial difference between correlation and causation. Thus, several human studies have performed MR studies to reveal the causal effect e.g., of risk factors on diseases based on genetic variants. To date, no MR study has investigated the causal effect of MY on health traits in dairy cattle. Thus, we performed a MR analysis with MY as the exposure variable and the health traits as the outcome variables. To do so, we applied a method that uses summary statistics and that removes horizontal pleiotropic variants [15]. In addition, we estimated genetic correlations using the 50K chip data to obtain observational estimates of the connections between traits and to compare them with the estimates of causal effects from the MR analysis. Of seven trait-combinations, we found six significant genetic correlations as well as five nominal and two experiment-wide significant causal associations. The latter were between the traits with the highest genetic correlation. All estimates had a negative sign. Moreover, we identified six pleiotropic variants but found no substantial impact of these variants on the estimates of causal effects.

### Biology

All the genetic correlations, except for that between MY and DD, were significant. On the opposite, only two experiment-wide significant causal associations were found, which were between MY and PH and MY and MAS. Interestingly, these traits were also those with the strongest genetic correlation with MY (Table 2). Regarding the causal effect of MY on the health traits, it is possible that the connection between the traits is mediated by another trait via vertical pleiotropy. Here, it is important to mention that, in contrast to horizontal pleiotropy, vertical pleiotropy does not result in instrumental invalidity. This is because in the case of vertical pleiotropy, the mediating variable is on the same causal pathway as the exposure and the outcome variables [16]. A putative mediating variable is the negative energy balance (NEB), which is a physiological imbalance that occurs within two to three months after calving [34, 35]. In this period, the immune system of the animals is suppressed, the level of immunocytes is decreased and the level of inflammatory cytokines and reactive oxygen species that damage cellular tissue is increased [36]. This shift in the immune system can make individuals susceptible to infectious diseases, such as MAS or infectious claw diseases. Van der Spek et al. [37] defined DD, PH and IH as infectious and LAM, WL, and CU as non-infectious claw diseases. Indeed, the incidences of DD and PH were found to be highest in primiparous cows during the first months after calving [38]. Thus, we assume that the experiment-wide significant causal associations between MY and MAS and between MY and PH and the almost experiment-wide significant one with DD in this study might be due to the NEB as mediating variable. In addition, a nutritional imbalance, such as the NEB, is characterized by an insufficient intake of minerals and vitamins. This results in the weak formation of the claw horn, which makes the individual susceptible to infectious diseases [38]. Although IH also belongs to the group of infectious diseases, we did not find a significant causal effect of MY for this claw disease, neither experiment-wide nor nominal. A possible explanation would be that a genetic predisposition is causing IH rather than a causal effect of MY. This is based on the results of previous studies, which found chromosomal regions [39] and a missense mutation on BTA8 [40] that are associated with IH. Another potentially mediating variable is the gut microbiome. An effect of the gut microbiome on the immune system [41], udder [42, 43] and claw health [44] has already been mentioned by several studies. In addition, Monteiro et al. [45] reported an association between the level of milk production and the microbiome of the rumen as well as of the lower gut. Thus, it is possible that an elevated level of milk production leads to a change in the gut microbiome, which in turn could result in a deteriorated health status of the cows. Finally, a third variable that might mediate the effect of MY on MAS is the milking speed (MS). Several studies mentioned a positive genetic correlation between MS and MY [46–48] but also an unfavorable genetic correlation between udder health and MS [48–51]. Combining these findings, we suggest that a higher MY evokes an increased MS and thereby also an increased risk to develop mastitis.

We did not observe a large difference between the HEIDI and the noHEIDI analysis. This was surprising as horizontal pleiotropy has been reported to be common in humans and livestock [52, 53]. An explanation for our findings might be that only top associated variants were used as IV, which might not be the horizontal pleiotropic variants in this case. However, six horizontal pleiotropic variants were detected and removed by the HEIDI method, which were mostly (four out of six variants) on BTA6 (Table 3). Two of them are either located within (BTA6:87304531, MY-MAS) or nearby (BTA6:87719361, MY-DD) genes that have previously been identified as quantitative trait loci (QTL) for longevity [54, 55]. In addition, the variant on BTA6 at 87,304,531 bp is located within the *NPFFR2* gene, which is a QTL for mastitis resistance [56], somatic cell score, milk, and protein yield [57]. This variant is also located nearby the *GC* gene, which has a horizontal pleiotropic effect on milk production and mastitis [58]. A study on resilience indicator traits, conducted by Chen et al. [24], revealed two regions on BTA6 showing a noteworthy association with some resilience indicator traits. These regions are located close to the horizontal pleiotropic variants on BTA6. Moreover, the variant on BTA19 at 17,893,592 bp that affects MY and MAS, in our study, is located nearby the *RHBDL3* gene, which was mentioned to have an influence on a wide range of human diseases like cancer or inflammatory diseases [59]. These results from previous studies support our findings that the horizontal pleiotropic variants, which were identified by the HEIDI method, have an effect on MY and several health traits.

### Methodology

Another popular method to assess causality are structural equation models (SEM) [60] that enable the identification of causal relationships among traits and allow for the prediction of one trait by another “upstream” causal variable [61]. Recently, they have been extended to GWAS-SEM, where the total genetic effects for one trait are separated into direct and indirect genetic effects, i.e., genetic effects that are mediated by other upstream variables [62–64]. Thus, SEM provide detailed information about causal associations among traits. However, they are sensitive to erroneous model specifications, computationally very complex and require a temporal sequence of traits, i.e., that one trait has to occur prior to the other one [19]. Thus, we did not apply a SEM in this study.

As for all statistical analyses, a MR also relies on untestable assumptions and thus, the inference that one can draw depends strongly on the plausibility of the assumptions [6]. In the case of a MR, caution has to be paid to the choice of the variants as IV, according to the three initially mentioned assumptions. In order to meet the relevance assumption that relates to the association between a variant and the exposure trait, we used only strongly associated variants (${p}_{GWAS, MY}< {5*10}^{-8}$) as IV for our analyses. It has been shown that using these variants ensures the absence of inflation in the test statistics under the ${H}_{0}$ that ${b}_{xy}=0$ [15]. In addition, applying only top-associated variants also helps to avoid weak instrument bias, i.e., that IV have a weak effect on the exposure variable, which would result in low power of the MR analysis. Moreover, a weak instrument bias is also attenuated by applying multiple variants instead of only one, since they are more likely to explain a larger proportion of the genetic variance [16]. Because of this, we applied summary statistics for our analysis. Nevertheless, quantitative traits are typically polygenic in nature, i.e., they are influenced by many variants that all have small effect sizes. Thus, applying only the top-associated variants as IV neglects this polygenicity, which might hamper the detection of causal exposure outcome associations [65]. Indeed, it is possible that the absence of causal associations among all the traits in our analysis is caused by the stringent threshold that we set for the definition of IV. However, if a variant has a larger effect on the outcome variable and a smaller effect on the exposure variable, we assume that this variant is less likely suitable for indicating causality. Instead, this variant is more likely having a horizontal pleiotropic effect on both the exposure and the outcome variables. Applying horizontal pleiotropic variants as IV would violate the third IV assumption, i.e., the exclusion restriction assumption [16]. Hence, we decided to apply only the top-associated variants as IV. Moreover, the power of a MR analysis depends strongly on the power of the underlying GWAS, the summary statistics of which are used in the MR. Thus, our aim was to apply a large sample size to our GWAS which results in greater power to detect significantly associated variants. It has also been shown that the power of a GWAS benefits from the application of sequence rather than SNP chip data [66]. On the downside, an increased sample size can also evoke inflated false positive associations [67–69]. A metric to identify genomic inflation is the genomic inflation factor, where a value below 1 points to a deflation and a value above 1 points to an inflation in the GWAS signals. Acceptable values are below 1.5 [32]. In order to avoid both, a weak instrument bias and an inflation in the MR estimates caused by an inflated summary statistic, we decided to proceed with the summary statistics for each trait that had a genomic inflation factor closest to 1. Hence, we applied the summary statistics from the MLMA method for the health traits, whereas this method was deflated for MY. By applying the LOCO method, we found a strong inflation for MY, which made it necessary to apply the PC_CHR method to generate a summary statistic with a suitable genomic inflation. Including PC as covariates in addition to the GRM in GWAS has already been implemented in other studies on livestock to mitigate the effect of population stratification as a strong confounding bias [26, 32, 33].

An important aspect is the interpretation of the MR results in our study. MR estimates are meant to deliver credible causal associations between exposure and outcome variables [15] and relate to genetically-induced effects of the exposure on the outcome variable [6]. Genetic variants are fixed at conception. Hence, they indicate long-term effects of elevated levels of the exposure variable and cannot be understood as alterations in the outcome that one can expect by an intervention on the exposure variable at a specific time in life [6, 8, 16]. This hampers a quantitative inference from levels of e.g., LDL or vitamin D that are typically measured at a certain time in life. However, we used DRP as phenotypes in our study, which do not relate to a specific time. Thus, the causal effect of MY on MAS ${\widehat{b}}_{xy}=$ − 0.1309 indicates that an increase by one standard deviation (SD) of the DRP for MY results in a decrease by 0.1309 SD of the DRP for MAS. Thus, it is important to keep in mind that MR relies on untestable assumptions and the credibility of the results depends strongly on the plausibility of these assumptions. As already mentioned, our aim was to pay attention to fulfilling these assumptions, e.g., by applying strongly associated genetic variants as IV or by removing horizontal pleiotropic variants. However, we believe that the inference one draws from ${\widehat{b}}_{xy}$ should be understood rather qualitatively than quantitatively, also because the DRP themselves are obtained from estimations that rely on statistical assumptions.

Several methods to perform a MR analysis have been developed and are reviewed for example in Davies et al. [16], Slob and Burgess [70], and Burgess et al. [6]. They differ e.g., in the assumptions they make about instrumental invalidity and the way they account for horizontal pleiotropy. It has been mentioned that causal effects can be verified by applying various methods [6, 70]. However, we decided not to apply any other method than the GSMR because (1) it accounts for LD among variants, (2) it has been found to be not or only little affected by undetected horizontal pleiotropy, and (3) it outperforms other methods regarding the power to detect causal effects. The latter feature is based on the fact that GSMR takes the sampling variance in ${\widehat{b}}_{zy}$ and ${\widehat{b}}_{zx}$ into account as opposed to other methods [15]. Hence, we aimed at verifying the causal effects that were found in this study by means of a reverse MR analysis using the health traits as the exposure and MY as the outcome variable. Thus, the causal effects of MY on the health traits would have been verified if the corresponding reverse causal effects were negligible. Unfortunately, it was not possible to perform a reverse MR analysis because too few, i.e., less than ten, IV remained after filtering for LD among the variants. Thus, further studies are required to validate the causal association of MY especially with MAS and PH in other datasets.

Finally, a limitation of our study is that it is a one-sample MR, which means that the summary statistics for the outcome and exposure variables were generated using the same sample. This makes environmental confounding more likely to occur. Environmental confounding implies that genetic causal associations between the exposure and outcome variables are correlated in the direction of a confounding association between these two, which induces an increased type I error rate of the MR estimates [6]. However, whereas human geneticists can benefit from a huge range of publicly available summary statistics, this is not the case in animal genetics. Hence, we decided to proceed with the summary statistics that we generated ourselves and aimed at minimizing confounding by applying DRP in the GWAS. The DRP originated from the national breeding value estimation in Germany, where environmental factors are corrected for by using fixed effects as well as by applying a test-day random-regression model for MY [71]. Another limitation is that a one-sample setting might suffer from the effect of the winner´s curse, i.e., that genetic associations tend to be overestimated in the dataset in which they were first discovered. This again might induce a weak instrument bias if the true association is much lower than the over-estimated one [6]. In general, these limiting factors can cause an inflation in MR estimates. However, we observed that only two of seven health traits showed an experiment-wide significant causal association with milk yield and believe that this confirms the credibility of our results.

## Conclusions

Exploiting the large sample size and sequence data to achieve sufficient statistical power in this study enabled us to more deeply investigate the genetic connection between milk yield and health traits in German Holstein. Regarding the generation of the summary statistics, our results confirm the need to include PC in the GWAS for milk yield to avoid substantial genomic inflation caused by population stratification. Moreover, an experiment-wide significant negative causal association was found between MY and digital phlegmon as well as between MY and mastitis and a nominal significant negative causal association between milk yield and almost each health trait except for interdigital hyperplasia and laminitis. This indicates the long-term negative impact of a genetically driven high milk yield on animal health. However, this causal association still requires to be validated using other samples. Not only the causal associations but also the genetic correlations were all negative. We assume that the causal association between MY and these health traits might be due to another mediating variable, such as the NEB. In the MR analysis, horizontal pleiotropic variants that affected both, MY and the health traits, were identified and removed using a filter step that is implemented in the GSMR method, which we used for our analysis. Interestingly, the difference between estimates of causal effects with and without horizontal pleiotropic variants was negligible. Our findings support the potential of investigating causal associations between economically important traits in livestock by applying a MR analysis in order to unravel these relationships between traits in more detail, and to better differentiate between their causal associations, the effect of horizontal pleiotropic variants affecting both traits and linkage between QTL.

## Data Availability

Restrictions apply to the availability of the genotype and phenotype data analyzed in this study since they are property of the German Holstein breeding organisations organized within the Bundesverband Rind und Schwein e.V.. We thank all participating organisations. Thus, the data have commercial value and are not publicly available. The corresponding author can be contacted for a reasonable request.
